# Video-assisted thoracoscopic blebotomy for spontaneous pneumothorax treatment using the no-knife endoscopic stapler

**DOI:** 10.1007/s00383-026-06391-w

**Published:** 2026-03-25

**Authors:** Sunghoon Kim, Gabriella Grisotti, Olajire Idowu

**Affiliations:** 1 Division of Pediatric Surgery, University of California San Francisco Benioff Children’s Hospitals, Oakland, CA USA; 2 Division of Pediatric Surgery, University of California San Francisco Benioff Children’s Hospitals, San Francisco, CA USA; 3 Division of Pediatric Surgery, University of California San Francisco Benioff Children’s Hospitals, Oakland, CA USA

**Keywords:** Spontaneous pneumothorax, Blebotomy, No-knife endoscopic stapler, Pleurodesis, Thoracoscopic blebectomy, Video-assisted thoracoscopic surgery

## Abstract

**Background:**

A standard treatment for pneumothorax due to bleb rupture is thoracoscopic blebectomy, often combined with pleurodesis to reduce recurrence risk. However, pleurodesis can cause significant postoperative pain. This study evaluates the efficacy of the no-knife endoscopic stapler blebotomy without pleurodesis using video-assisted thoracoscopic surgery (VATS) for spontaneous pneumothorax.

**Methods:**

We conducted a retrospective chart review of patients treated with no-knife endoscopic stapler blebotomy for spontaneous pneumothorax at UCSF Benioff Children’s Hospital Oakland from 2020 to 2024. The procedure involved using the no-knife endoscopic stapler for blebotomy without performing pleurodesis. Post-procedure air leaks were monitored with a chest tube. We analyzed postoperative chest tube duration, pain levels, and recurrence rates.

**Results:**

The study included eight male patients with a median age of 17 (range 15-19). Apical blebs were identified thoracoscopically in all patients and stapled off using the no-knife stapler. No intraoperative or postoperative complications occurred. The median postoperative chest tube duration was 2 days (range 1-3). The mean pain score on postoperative day 1 was 1.3 (range 0-10). Over a median follow-up of 24 months (range 6-36), no pneumothorax recurrence was observed.

**Conclusion:**

No-knife endoscopic stapler thoracoscopic blebotomy is a safe and effective procedure. Avoiding pleurodesis reduces postoperative pain and morbidity.

**Level of evidence:**

Level 4, Observational study.

## Introduction

Spontaneous pneumothorax (SP) is characterized by the accumulation of air in the pleural space, leading to lung collapse. SP is classified into two types: primary spontaneous pneumothorax, which occurs in individuals without underlying lung disease, typically young, tall, thin males who develop apical lung blebs; and secondary spontaneous pneumothorax, which appears in patients with pre-existing lung conditions such as chronic obstructive pulmonary disease, cystic fibrosis, or interstitial lung disease. Initial SP episodes can be managed with observation, oxygen therapy, aspiration of pleural air, or chest tube placement. However, surgical intervention is warranted for recurrent pneumothorax, persistent air leaks, bilateral pneumothorax, or when occupational risks (e.g., pilots, divers) make the risk of recurrence life-threatening [1].

The most common surgical treatments for SP include video-assisted thoracoscopic surgery (VATS) blebectomy, which removes the blebs responsible for the air leak, and pleurodesis, which induces an inflammatory reaction to adhere the lung to the chest wall and prevent recurrence [2]. Although effective, pleurodesis is associated with significant postoperative pain and longer recovery times. An alternative method that eliminates the need for pleurodesis could significantly reduce postoperative morbidity, and the length of stay would be reduced.

This study evaluates the efficacy and safety of no-knife endoscopic stapler thoracoscopic blebotomy for the treatment of SP. We hypothesize that if the stapled bleb lung tissue remains in situ, it will act as a natural adhesive force, causing innate pleurodesis.

## Methods

**Fig. 1:**
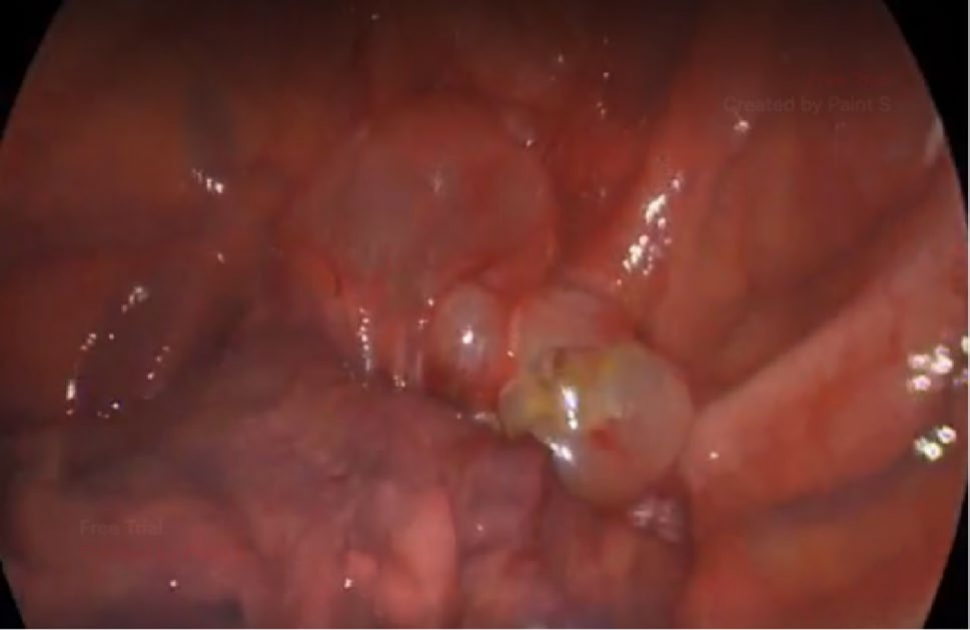
A large apical bleb is demonstrated

A retrospective review was conducted on patients who underwent non-cutting endoscopic stapler VATS blebotomy for primary SP at the UCSF Benioff Children’s Hospital Oakland from 2020 to 2024. Clinical and radiological evaluations, including serial chest X-rays and pre-operative chest CT scans, confirmed SP and identified lung blebs. Patients included those with previous episodes of pneumothorax or persistent chest tube air leaks necessitating surgical intervention (Fig. 1).

**Fig. 2:**
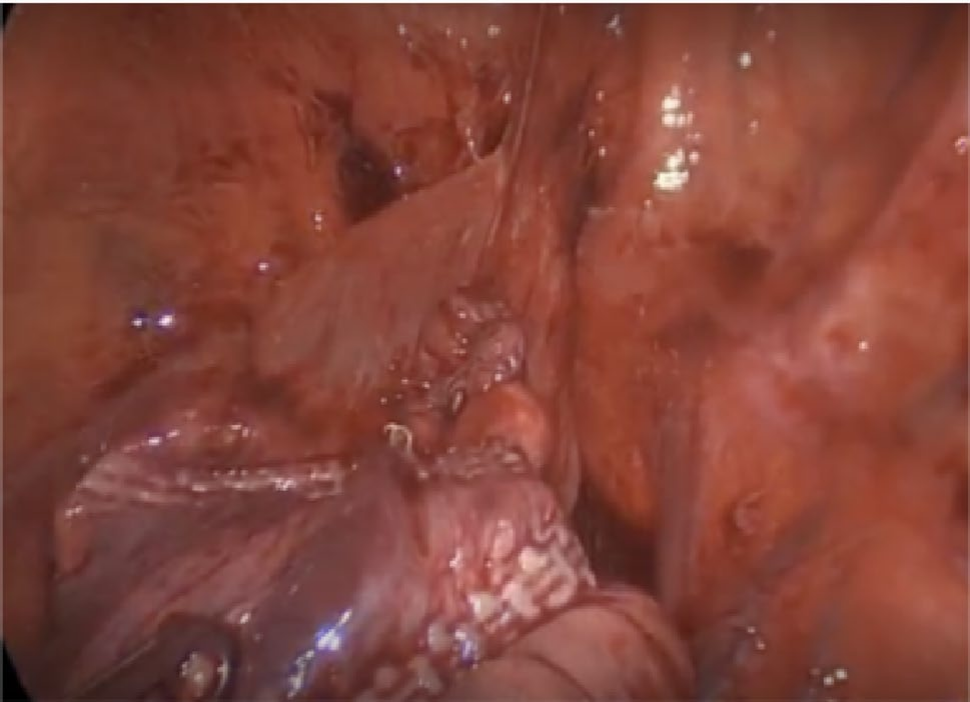
A no-knife endoscopic stabler is used to perform the blebotomy. More than one firing of the stapler was needed to occlude the bleb from the lung

The standard VATS procedure was performed as follows: Patients were placed under general anesthesia with single lung ventilation in a lateral decubitus position, affected side up. Three ports were used: one 12 mm and two 5 mm, with local anesthetic Ropivacaine for incisional pain control. Ports were strategically placed in the posterior lateral chest to triangulate the lung apex. Carbon dioxide insufflation at 5 mmHg further suppressed the lung. A 30-degree thoracoscope was used for visualization. Upon identifying the apical lung bleb, a no-knife endoscopic stapler (ENDOPATH ETS 45 mm Articulating Linear Cutter Stapler - Ethicon Inc., Raritan, NJ, USA) was used to staple the lung tissue below the bleb, leaving the stapled tissue in situ. Multiple applications of the stapler were made for large or numerous blebs, occasionally requiring repositioning of the 12 mm port. Despite being labeled a cutter, the stapler does not cut the tissue; it applies six rows of staple lines. The lung apex was tested for air leaks using saline immersion, followed by placement of a 16-Fr chest tube connected to Pleurovac for postoperative air-leak monitoring. Daily chest X-rays were taken until chest tube removal. Port sites were closed in layers, and the patient was extubated and transferred to recovery (Fig. 2).

Outcome measures included recurrence rate, hospitalization duration (measured by chest tube duration), postoperative day 1 pain levels while the chest tube was on suction, as recorded by nursing staff, and any intraoperative or postoperative complications, such as prolonged air leaks. Post-discharge follow-ups occurred in person at 2 weeks, then biannually by phone. Chest X-rays were obtained if patients reported dyspnea or chest pain.

Ethical approval for the study was obtained from the UCSF Institutional Review Board.

## Results

The study included eight male patients with a median age of 17 (range 15–19). The patient’s average weight was 58 kg. Two out of 8 patients had previous treatment for spontaneous pneumothorax with thoracoscopic blebectomy and pleurodesis (one had doxycycline and another had mechanical pleurodesis). Six patients were newly diagnosed with SP. Apical blebs were identified in all patients thoracoscopically. Three patients had multiple large blebs requiring 2–3 staple cartridges, and the remaining five had a single bleb treated with a single staple fire. No intraoperative complication was observed. No patients had a prolonged air leak post-operation. The median postoperative chest tube duration was 2 days (range 1–3). Over a median follow-up of 24 months (range 6–36), no recurrence of pneumothorax was documented. Patients reported minimal postoperative pain. The mean Pain Numeric Rating Scale score (0–10) on post-operative day 1 was 1.3 (range 0–4). Post-operatively, all patients received ketorolac 30 mg intravenously for 24 h for pain control and were prescribed as-needed IV morphine (third line), oxycodone (second line), and acetaminophen (first line) for post-operative pain management. The review of the pain medication intake showed no use of morphine for all eight patients. Patients received either acetaminophen or oxycodone as needed. All patients were discharged home on only acetaminophen or ibuprofen and had a quick return to normal activities.

## Discussions

Pleurodesis involves inducing an inflammatory reaction between the pleural layers, causing them to adhere and obliterate the pleural space to prevent lung collapse. It can be achieved mechanically through pleurectomy, pleural abrasion, or chemically by instilling a sclerosing agent like talc or doxycycline. Pleurodesis is highly effective in preventing pneumothorax recurrence, with a recurrence rate of 2% [3]. However, it is associated with significant postoperative pain due to pleural inflammation. Extended pain management may be required, and complications can include acute respiratory distress syndrome, infection, and chronic pleuritic pain. Additionally, extensive pleurodesis is irreversible, complicating future thoracic surgeries.

This study suggests no-knife endoscopic stapler blebotomy is a safe and effective alternative for preventing recurrent pneumothorax. The technique demonstrated no recurrences over a median follow-up of 24 months, no postoperative complications, and favorable recovery metrics. Unlike cutting staplers, the no-knife stapler lays down six rows of staple lines, potentially reducing prolonged air leaks. We conjecture that the in situ bleb tissue undergoes necrosis, promoting inflammation at the chest apex and causing natural pleurodesis. There is a legitimate concern that leaving a devitalized tissue may lead to fever, infection, or cause pleuritic chest pain. However, these complications were not observed in any of the patients.

The results have significant clinical implications for managing spontaneous pneumothorax, especially for young, healthy individuals with primary SP who wish to avoid the morbidity associated with painful pleurodesis. This technique may also benefit patients with complications or recurrences from traditional surgical methods. Reduced chest tube placement duration and shorter hospital stays can lead to cost savings and improved resource utilization. Enhanced recovery protocols can further improve patient outcomes and satisfaction. Exploring this technique’s use in secondary spontaneous pneumothorax and complex cases like lung reductions for emphysema could expand its applicability.

While promising, this study has limitations. The retrospective design may introduce selection bias and limit the generalizability of the findings. Prospective randomized controlled trials are needed for more robust evidence of this technique’s efficacy and safety. The small sample size limits the statistical power and ability to detect rare complications or outcome differences. Larger studies with diverse patient populations are necessary to validate these results. Although the median follow-up of 24 months is adequate for short- to mid-term outcomes, longer follow-up is needed to assess the procedure’s durability and late recurrence risk.

In conclusion, no-knife endoscopic stapler blebotomy represents a promising alternative to traditional methods for preventing recurrent pneumothorax. This minimally invasive technique avoids pleurodesis and appears to reduce postoperative pain, shorten hospital stays, and improve recovery outcomes. The absence of recurrences in our study underscores its potential effectiveness. Further research, including larger prospective studies and long-term follow-up, is warranted to confirm these findings and establish this technique as a standard treatment for spontaneous pneumothorax.

## Data Availability

No datasets were generated or analysed during the current study.

## Abbreviations

VATS: Video-assisted thoracoscopic surgery
SP: Spontaneous pneumothorax
